# Notes from Past Meetings of the Lippe Society

**Published:** 1890-08

**Authors:** 


### NOTES FROM PAST MEETINGS OF THE LIPPE SOCIETY.

Dr. Carleton Smith spoke of the difference between Mercurius and Podophyllum. To be certain that Merc. is indicated, the patient should be requested to put out his tongue; biting upon it slightly causes the well-known impression of teeth. Merc-prot. has for key-note yellowness at root of tongue.

Podophyllum has flatulence on right side of abdomen only, with palpitation of the heart. This is peculiar to Podoph. Another peculiar symptom of Podoph. is falling of the womb, with pain in the back, which is worse from leaning over the wash-tub.

The Merc. patient, when using an outside water-closet, is made worse by taking cold from the draughts of air.

Dr. Fellger—Mercury is a remedy that affects the liver more than any other drug. After death the reguline metal may be found in the liver of those who have taken it. Silver is found in the heart and lead in the brain.

A man with yellow fever was treated in an old-school hospital with twenty-grain doses of Mercury. When, some time after this, Dr. Fellger made a post-mortem examination, he found a large quantity of Mercury in the liver. The other organs were healthy. Dr. Fellger also said that those who have prolapsus recti are usually affected with elongated uvula. In the pathogenesis of Mercury are recorded falling of uvula and prolapsus of the rectum. Natrum-muriaticum is a good remedy where a patient with an affection of the back can stoop over readily, but it hurts him to straighten himself again.

In gonorrhœa, the slightest inflammation is much increased by getting cold from the draughts of outside water-closets. The inflammation may thus be made to extend to the prostate gland and the bladder. Hence, in all cases of gonorrhœa he makes particular inquiry as to the kind of water-closet used. If the patient has access to outside water-closets only, he orders him to wrap the genitals in a towel. This is one of the main difficulties in treating gonorrhœa. In gonorrhœa, as in all other affections, it is necessary to examine into the entire condition. Thus, taking as a guide, with other symptoms, furuncles on the lower lip, he gave Petroleum, and effected a rapid cure in a case of severe gonorrhœa.

In reply to a question, Dr. Fellger said two capital remedies for sleeplessness of children at night are Coffea and Opium. Coffea, restlessness then sleep; Opium, sleep then restlessness.

Dr. Carleton Smith said, Where the baby sleeps all day and cries all night, give Lycopodium. In these cases there is often the red sand in the urine.

Dr. Fellger—Sulphur has the symptom, urine clear and colorless as water. In such cases the child is dangerously ill.

Continuing about Mercury, Dr. Fellger thought no drug was so abused by the old school. It is found not only in the liver but in the bones. In a museum in Strasburg he had seen a room filled with bones of those who had been mercurialized. The metallic mercury was plainly visible. There were skulls where section of the diploœ showed large accumulations of Mercury.

